# Examining the Relationship of Information and Communication Technologies Use and Reading Literacy: A Moderated-Mediation Analysis of Metacognition Across Information and Communication Technologies Use Intensity

**DOI:** 10.3389/fpsyg.2022.916497

**Published:** 2022-07-06

**Authors:** Miaoyun Li, Meiqian Wang

**Affiliations:** National Engineering Research Center for E-Learning, Central China Normal University, Wuhan, China

**Keywords:** reading literacy, ICT use, metacognition, ICT use intensity, moderated mediation analysis

## Abstract

The use of information and communication technologies (ICT) is increasingly becoming prevalent among students, both at home and school. While inconsistent results were found for student ICT use and reading literacy, this study attempted to explain these ambiguous links with the moderation of ICT use intensity and mediation of metacognition. Three moderated mediation models for each type of ICT use (at home for entertainment activities and for schoolwork, as well as at school) were analyzed using a Hong Kong sample taken from the Programme for International Student Assessment (PISA) 2018 data pertaining to 5180 15-year-old students from 152 schools. A dynamic effect pattern was found for the links of all ICT use types and reading literacy with the increasing intensity of ICT use, which begins with a positive effect followed by a decrease to less positive, then turns to fluctuating negative and finally ends up with a stable negative effect. But the dominant effect varies across ICT use intensity, which result in different overall effects of three ICT use types. In addition, all three aspects of metacognition showed a profound negative mediation on links of intensive and excessive ICT use with reading literacy, and a less positive mediation for limited ICT use. The metacognition of assessing credibility showed a more important role than summarizing, which was followed by understanding and remembering. In light of the findings, the study recommended that more metacognitive scaffolds should be developed for students with intensive or excessive ICT use, so as to alleviate the side effects of ICT use on their reading literacy.

## Introduction

With the digitalization of modern society, information and communication technology (ICT) resources have become widely available to students both at home and school. ICT use has become prevalent among students across all levels, from the primary level to universities. Several studies have indicated that the appropriate use of ICT tools offers numerous advantages in students’ social, psychological and cognitive development, such as stimulating student well-being, creativity, and motivation (Allegra et al., 2001; Tüzün et al., 2009; Orben and Przybylski, 2019; Rasheed et al., 2020; Huang et al., 2021), and allowing for greater interactivity, cooperation, and communication with their teachers and peers (Schulz-Zander et al., 2002; Koç, 2005; Li et al., 2019). It is also reported to improve students’ academic performance (Borgonovi and Pokropek, 2021; Sapci et al., 2021; Kong et al., 2022). Thus, many programs have been implemented to provide the necessary infrastructure and resources for the integration of ICT into student life both at home and school.

However, ICT tools are also frequently cited to cause problems, such as attention deficiency and addiction to ICT (Papanastasiou et al., 2005; Turel and Toraman, 2015; Kates et al., 2018). In addition, excessive exposure to ICT resources and tools would lead to the use of resources with poor reliability (Gómez-Fernández and Mediavilla, 2021). According to Gómez-Fernández and Mediavilla (2022), students’ frequency of ICT use matters for teachers, and that they are more likely to use ICT in classes if students frequently use ICT at home, which would further increase student’s ICT use intensity, and then result in serious consequences on student psychological and social development, as well as their academic performance and acquisition of skills (Gómez-Fernández and Mediavilla, 2021). For example, Yunus et al. (2013) demonstrated that the use of ICT tools could cause lackadaisical attitudes among students whereby they do not take their work seriously. Additionally, developing reading skills by scrolling through a page on the computer screen could lead to an accelerated but superficial and inaccurate understanding of the content.

These potential advantages and disadvantages have raised debates on the effective implementation of ICT to facilitate the learning process (Gómez-Fernández and Mediavilla, 2021). This study attempted to explore this issue from the perspective of the relationship between student ICT use and reading literacy, as student reading literacy is the basis for acquiring knowledge and learning for other academic subjects (Cunningham and Stanovich, 1998; Borgonovi and Pokropek, 2021). A section of the literature has explored the association between student ICT use and reading literacy. Results of these associated studies reported positive associations coexisting alongside an absence of significant associations or negative associations. For example, Gómez-Fernández and Mediavilla (2021) demonstrated that student ICT use at home for leisure activities seems to be positively linked with reading literacy, while higher ICT use at home for schoolwork and ICT use at school is linked with lower reading literacy. Some studies have indicated a quadratic relationship between student ICT use at home and their reading literacy, implying that students with moderate use of ICT at school or outside school for schoolwork had the highest reading scores (Gubbels et al., 2020; Borgonovi and Pokropek, 2021). This indicates that the association of student ICT use and reading literacy varies with the increasing intensity of ICT use. However, there are no studies that explicitly explore this issue with the additional variable of ICT use intensity. Thus, the first aim of the present study is to confirm this by exploring whether student ICT use intensity moderates the relationship between ICT use and reading literacy, which can help us to further understand this relationship.

As negative effects were more likely to be found in instances of frequent or excessive ICT use, there is a need to analyze the negative effects of ICT use on academic performance in depth to identify the mechanism of these negative effects. Therefore, this study attempts to address this to help educational policies overcome these side effects by exploring the mediation of metacognition on the relationship of ICT use and reading literacy. Metacognition is a malleable variable, which can be enhanced through teaching and supportive classroom practices (Brozo and Simpson, 2007; Guthrie et al., 2013). Thus, exploring the role of metacognition in ICT use and reading literacy can provide insightful information for decision-makers on how to implement ICT to improve student reading literacy. Studies by Lee and Wu (2013); Wu (2014), and Chen et al. (2021a) showed that adolescent social media use is closely linked with their metacognition, which has also been proven to be a mediator in the relation between social media use and reading literacy. However, in these studies, social media use only refers to recreational use, such as reading news online, and not schoolwork. Therefore, the second aim of present study is to extend this line of inquiry to explore whether and how metacognition mediates the effects of different types of ICT use on reading literacy. Taking into consideration the mixed associations of ICT use with reading literacy, we would like to further explore whether this mediation varies across ICT use intensity.

This research is innovative and contributes to the previous literature for the following reasons. On one hand, this study attempts to confirm that student ICT use intensity moderates the links of three types of ICT use and their reading literacy, which will make up for the limited literature on the analysis of ICT use intensity’s impact on the relationship between student ICT use and reading literacy. This would help to better explain the paradox findings of previous studies on the relationship between ICT use and reading literacy. On the other hand, this study explores the role of metacognition in the relationship between student ICT use and reading literacy, the results can help to provide insightful information on the instructional strategies to alleviate the side effects of ICT use on reading literacy.

In sum, to fill the research gap in current studies and explore the processes involved in the association between ICT use and reading literacy, this study attempted to explore the following two research questions:

(1) How does the relation between student ICT use, including ICT use at home for entertainment activities (ENTUSE) and for schoolwork (HOMESCH) as well as ICT use at school in general (USESCH), and reading literacy vary across ICT use intensity?

(2) How does student ICT use intensity moderate the mediation of metacognition on the relationship between student ICT use and reading literacy?

## Literature Review

With the increasing integration of ICT into education, its scope is expanding, and its links with reading literacy are also changing. This study attempts to explore these changing links.

### Information and Communication Technologies Use and Reading Literacy

The research on ICT use has expanded from its original simple introduction as one type of ICT device (e.g., computer, broadband, and laptops), to the specific application of one or a number of ICT hardware or software (e.g., learning management system); furthermore, three types of ICT use are defined in general nowadays: ENTUSE, HOMESCH, and USESCH.

Several studies have explored the relation between student ICT use and reading literacy, and the results were ambiguous. Some researchers examined the relationship of ICT use and reading literacy based on the Programme for International Student Assessment (PISA) dataset. Fuchs and Woessmann (2004) found positive effects of computer use on student reading scores in the data of all countries participating in PISA 2000. The positive effects were also found in literature that have explored specific ICT tools’ influence on students’ learning. For example, studies by Barrow et al. (2009) and Belo et al. (2016) found that students who used computer programs performed better in reading than those who were exposed to traditional teaching methods. Grimes and Warschauer (2008) and Lai et al. (2015) indicated that the use of ICT devices in the classroom can improve student academic performance in English and math at the end of 2 years of their participation. Rouse and Krueger (2004) observed limited improvement in reading skills due to the implementation of computer programs. Meanwhile, some negative effects were also found. Mora et al. (2018) found that the introduction of the One Laptop per Child program by the Catalan government in Spain had a negative impact on student performance in language (Catalan, Spanish, and English) learning.

Significantly, the effects of ICT use on reading literacy varied with the use purpose. Falck et al. (2018) suggested that the use of computers to look up information increased students’ reading literacy, while using them to practice skills decreased reading literacy, with the compensation of these two effects resulting in overall null effects. Hu et al. (2018) found that student ICT academic use negatively correlated with their reading literacy, while ICT entertainment use positively correlated with reading literacy. Regarding the relationship between ICT use for schoolwork both at home and school, mainly negative effects were found based on the analysis of PISA data in the large majority of countries (Biagi and Loi, 2013; Petko et al., 2017; Hu et al., 2018; Vazquez-Lopez and Huerta-Manzanilla, 2021) with only a few countries or samples showing positive or null effects.

### Moderation of Information and Communication Technologies Use Intensity

Most notably, Gubbels et al. (2020) found that the links between ICT use and reading literacy were not linear but followed an inverted U-shape, indicating that moderate but not excessive use of ICT was linked with the highest reading performance. Coincidentally, via quantile analysis, Gómez-Fernández and Mediavilla (2021) found that the magnitude of the negative effect of ICT use at school in general on reading literacy is always higher at the lower part of their ability distribution (25th and 50th percentile). In the context of home use, the U-shaped relationship has also been elucidated in previous studies. Agasisti et al. (2020) found that greater use of ICT at home, explicitly in relation to school-related tasks, was negatively linked with students’ test scores in reading. With regard to ENTUSE, frequent use was found to relate negatively to reading performance; the more students use ICT for leisure, the lower their reading performance (Steffens, 2014).

Based on these findings, this study hypothesized that the effects of ICT use on reading literacy may dynamically change with the intensity of ICT use. Low intensity ICT use produced more positive effects. With increasing familiarity with ICT tools, student attention may drift from learning to some degree, and thus, the effects of ICT use may decrease and show an overall less positive or even non-significant effect. If this issue is not addressed and students remain addicted to ICT tools, it may even lead to negative effects.

Thus, we hypothesize that the effect of ICT use on reading literacy was moderated by the intensity of ICT use. In this study, we aim to test this hypothesis for each type of ICT use separately when controlling for the other two types of ICT use. Thus, three sub-hypotheses were produced, which are presented in Table 1.

**TABLE 1:** The specific hypothesis of moderation analysis.

ID	Content of hypotheses
H1	The effects of ICT use on reading literacy was moderated by the intensity of ICT use.
H1a	The effect of ENTUSE on reading literacy would be moderated by the intensity of ENTUSE.
H1b	The effect of HOMESCH on reading literacy would be moderated by the intensity of HOMESCH.
H1c	The effect of USESCH on reading literacy would be moderated by the intensity of USESCH.

*ENTUSE, ICT use at home for recreational activities; HOMESCH, ICT use at home for schoolwork; USESCH, ICT use at school in general.*

### Moderated Mediation of Metacognition

We need to further explore the relation between ICT use and reading literacy to ascertain optimal procedures for educational policies and interventions. The second aim of this study tried to fill this research gap from the standpoint of mediation of metacognition.

Metacognition, also known as metacognitive strategies, is defined as “an individual’s ability to think about and control his or her reading and comprehension strategies” (OECD, 2019, pp152). The prominent metacognitive strategies in reading tasks include setting goals, adapting reading strategies corresponding to these goals, knowing how to summarize a piece of text or remember essential information, monitoring comprehension, and knowing how to address comprehension problems (OECD, 2019).

Several studies have found positive links between metacognition and reading literacy. It has been demonstrated that students trained using metacognitive strategy instruction showed better performance in reading comprehension than those who were only given conventional instruction procedures (Jairam and Kiewra, 2010). This positive link is more profound in online reading than in traditional print reading (Lee and Wu, 2013). Metacognitive strategies enable students to effectively interact with digital texts that are characterized by non-linear formats and hyperlinking structures, set up reading goals clearly, and deploy higher-order thinking strategies, such as analyzing, synthesizing, integrating, and evaluating information (OECD, 2019). In addition, it assists a skilled reader to evaluate the quality and appropriateness of the content (Liang et al., 2014).

Regarding the link between metacognition and ICT use, contradictory associations were found. The studies of Lee and Wu (2013) and Wu (2014) based on the PISA 2009 dataset found that metacognition was negatively linked with ICT use for social activities (e.g., collaborative online gaming, chatting online), but positively linked with ICT use for information-seeking activities (e.g., reading online news, search for practical information online). Combined with the positive link between metacognition and reading literacy, it was found that metacognition mediated the negative link of ICT use for social activities and reading literacy, but also mediated the positive link of ICT use for information-seeking activities and reading literacy (Lee and Wu, 2013). It was concluded that frequent online social/recreational activities can be detrimental to 15-year-olds’ knowledge of metacognitive strategies. Conversely, a positive effect of ICT use for recreation (e.g., chatting online, participating in social network) and digital reading literacy as well as a positive mediation of metacognition on this link was found in the studies of Chen et al. (2021a) based on the PISA 2018 dataset. It was explained that the ICT use 10 years ago required low mental efforts and their frequent use undermined student metacognitive strategies (Lee and Wu, 2013; Wu, 2014). In contrast, today’s wider range of ICT use activities required more reading experience and higher-order thinking strategies, which resulted in positive links between ICT use and metacognition (Coiro and Dobler, 2007; Kim et al., 2014). Consider these findings together, we can hypothesize that the appropriate ICT use can facilitate student knowledge of metacognitive strategies, while intense and even excess use may play a detrimental role.

**FIGURE 1:**
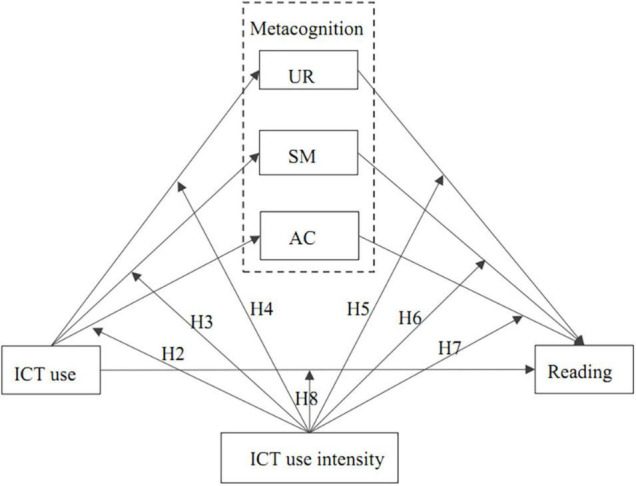
The conceptual model of moderated mediation hypothesizes. UR, Understanding and Remembering, SM, Summarizing, AC, Assessing Credibility.

In preceding studies, the role of metacognition was explored as a latent or observed construct, and its specific aspects were not explored further. Exploring specific aspects of metacognition can help us understand the functional mechanism of metacognition in depth, which can provide more information for practical classroom measures or interventions. Thus, differing from preceding studies, this study explored three aspects of metacognition, including understanding and remembering (UR), summarizing (SM), and assessing credibility (AC). In addition, preceding studies only explored ICT use for recreational activities, but not for schoolwork. With the considerable development rate of ICT tools, there is an urgent need to further explore how today’s different types of ICT use linked with their specific aspects of metacognition and reading literacy across ICT use intensity. Thus, our second research question is to explore how these three aspects of metacognition mediated the links between ICT use and reading literacy across ICT use intensity. The conceptual hypothesized model is described in Figure 1. We explored this for each type of ICT use separately. The specific hypotheses are presented in Table 2.

**TABLE 2:** The specific hypotheses of moderated-mediation analysis.

ID	Hypothesis
H2	The ICT use intensity moderated the link of ICT use with UR.
H2a	The ENTUSE intensity moderated the link of ENT with UR.
H2b	The HOMESCH intensity moderated the link of HOME with UR.
H2c	The USESCH intensity moderated the link of SCH with UR.
H3	The ICT use intensity moderated the link of ICT use and SM.
H3a	The ENTUSE intensity moderated the link of ENT with SM.
H3b	The HOMESCH intensity moderated the link of HOME with SM.
H3c	The USESCH intensity moderated the link of SCH with SM.
H4	The ICT use intensity moderated the link of ICT use and AC.
H4a	The ENTUSE intensity moderated the link of ENT with AC.
H4b	The HOMESCH intensity moderated the link of HOMESCH with AC.
H4c	The USESCH intensity moderated the link of USESCH with AC.
H5	The ICT use intensity moderated the link of UR with reading literacy.
H5a	The ENTUSE intensity moderated the link of UR with reading literacy.
H5b	The HOMESCH intensity moderated the link of UR with reading literacy.
H5c	The USESCH intensity moderated the link of UR with reading literacy.
H6	The ICT use intensity moderated the link of SM with reading literacy.
H6a	The ENTUSE intensity moderated the link of SM with reading literacy.
H6b	The HOMESCH intensity moderated the link of SM with reading literacy.
H6c	The USESCH intensity moderated the link of SM with reading literacy.
H7	The ICT use intensity moderated the link of AC with reading literacy.
H7a	The ENTUSE intensity moderated the link of AC with reading literacy.
H7b	The HOMESCH intensity moderated the link of AC with reading literacy.
H7c	The USESCH intensity moderated the link of AC with reading literacy.
H8	The ICT use intensity moderated the link of ICT use with reading literacy after controlling for the mediation of metacognition.
H8a	The ENTUSE intensity moderated the link of ENTUSE with reading literacy after controlling for the mediation of metacognition.
H8b	The HOMESCH intensity moderated the link of HOMESCH with reading literacy after controlling for the mediation of metacognition.
H8c	The USESCH intensity moderated the link of USESCH with reading literacy after controlling for the mediation of metacognition.

*ENTUSE, ICT use at home for recreational activities; HOMESCH, ICT use at home for schoolwork; USESCH, ICT use at school in general; UR, Understanding and Remembering of metacognition; SM, Summarizing of metacognition, AC, Assessing quality and Credibility of text sources of metacognition.*

## Materials and Methods

### Participants

Analyses were conducted on data for Hong Kong derived from PISA 2018.^1^ The target samples of PISA were 15-year-old students, and a two-stage probability proportional to size sampling procedure was used to select a representative sample (OECD, 2019). In the first phase, at least 150 schools were sampled in each country/region; in the second phase, about 42 15-year-old students within each school were randomly sampled to participate the survey. A total of 152 schools were selected from Hong Kong in China. After removing data with missing values listwise, a total of 5180 15-year-old students, who completed the student questionnaire and ICT familiarity questionnaire, were involved in this study. Among them, male students accounted for 49.40% (*N* = 2559) and female students accounted for 50.60% (*N* = 2621). Students of Grade 10 accounted for 68.88% (*N* = 3568), Grade 9 students accounted for 24.61% (*N* = 1275), and the remaining 6.51% students were from Grade 7, 8, and 11 (*N* = 337).

### Measures

#### Information and Communication Technologies Use and Information and Communication Technologies Use Intensity

Three types of ICT use, including ENTUSE for 12 items, HOMESCH for 12 items, and USESCH for 10 items, were measured by the ICT familiarity questionnaire in PISA 2018. Students were asked to indicate how often they used digital devices using a five-point Likert scale from 1 (never or hardly ever) to 5 (every day). Standardized composite variables provided by the PISA 2018 dataset were used. The internal consistency reliability (Cronbach’s alpha) was 0.84, 0.94, and 0.94 for ENTUSE, HOMESCH, and USESCH, respectively.

Because student original five ordinal responses on ICT use items are unevenly distributed, which indicates a too meticulous classification for five categories, present study attempted to categorize student ICT use intensity into four stages according to the frequency of ICT use. And from the least to the most intensity, these four stages were named as low use, intermediate use, intensive use and excessive use, respectively. The classification was done according to the 25, 50, and 75% percentile values of each type of ICT use score. Low use was defined by scores of lower than the 25% percentile value, intermediate use by scores between 25% percentile value and 50% percentile value, intensive use by scores between 50% percentile value and 75% percentile value, and excessive use by scores above the 75% percentile value. The distribution of ICT use intensity for each ICT use type is presented in Table 3.

**TABLE 3:** The distribution of ICT use intensity across three types of ICT use.

	Low use	Intermediate use	Intensive use	Excessive use
ENTUSE intensity	1279	1305	1320	1276
	24.69%	25.19%	25.48%	24.63%
HOMESCH intensity	1278	1305	1474	1114
	24.85%	25.19%	28.46%	21.51%
USESCH intensity	1285	1312	1300	1283
	24.81%	25.33%	25.10%	24.77%

*ENTUSE, ICT use at home for recreational activities; HOMESCH, ICT use at home for schoolwork; USESCH, ICT use at school in general.*

#### Metacognition

Metacognition is considered as a multi-component structure, which included UR, SM, and AC. Each aspect consists of a reading task and a set of strategies. A total of six reading tasks for UR, five tasks for SM, and five for AC were conducted. Students were asked to rate the strategies regarding their usefulness for solving the reading task on a six-point Likert scale ranging from 1 (not useful at all) to 6 (very useful). One example item for UR is that “I concentrate on the parts of the text that are easy to understand.” The final three indices were then scaled and standardized across OECD economies, with a mean of 0 and a standard deviation of 1 (OECD, 2019). The reliability for UR, SM, and AC is 0.79, 0.85, and 0.60, respectively.

#### Reading Literacy

In PISA 2018, reading literacy is defined as “understanding, using, evaluating, reflecting on and engaging with texts in order to achieve one’s goals, to develop one’s knowledge and potential and to participate in society” (OECD, 2019, p. 28). Following this definition, reading literacy was measured by three cognitive processes: accessing and retrieving information with a total of 25 items; forming a broad general understanding of the text and interpreting it with a total of 50 items; and reflecting and evaluating on the form of the text and its features with a total 25 items. In PISA 2018, ten plausible values which are imputation scores randomly from the posterior distribution of student reading literacy were allocated to each student. Plausible values provide some methodological advantages in comparison with classic item response theory, such as returning unbiased estimates of population performance parameters, percentages of student per proficiency level and bivariate or multivariate indices of relations between performance and background variables (OECD, 2009, p. 43). According to the Data Analysis Manual of PISA, the estimates and standard error for the analysis using one plausive value do not make any substantial difference with that using several plausible values when the sample size is large (OECD, 2009, p. 44). And due to the fact that the results generated from these ten plausible values were almost identical and for simplicity, the results for one random plausible value (PV10READ) were reported in this study with reference to some other studies (Trinidad, 2020; Chen et al., 2021b).

#### Covariates

A group of studies has indicated that student reading literacy has been influenced by their background information (e.g., gender; Borgonovi and Pokropek, 2021; Chen et al., 2021a; Kong et al., 2022). There is significant difference in reading literary between students with different levels of economic, social, and cultural status (ESCS; Chen et al., 2021a). Female students showed a higher score in reading literacy than their male counterparts (Chen et al., 2021a; Kong et al., 2022). Therefore, student’s gender and ESCS were considered as covariates and controlled in present study. Dummy variables were recoded for gender with 1 for girls and 0 for boys. In PISA 2018, the ESCS was constructed as the arithmetic mean of three indicators: highest parental occupation, parental education, and home possessions, after their imputation and standardization (Avvisati, 2020). And the ESCS score was reported after the transformation of having a mean of 0 and a standard deviation of 1 across senate-weighted OECD countries.

### Data Analysis

To answer the research question, a multilevel moderated mediation model with random intercept was constructed in this study. The multilevel method was used for the reason that the students involved in the present study were nested within schools (i.e., 5180 students from 152 schools) in nature. Even when our interested variable is the constructs of individual students, the multilevel modeling approach has to be used from a statistical perspective. In addition, the random effect analysis of variance showed that Intra-class Correlation Coefficients (ICCs) is 27.2% for reading literacy. It suggested that the differences of student reading literacy across schools should be taken into consideration, which indicated the justification of a multilevel regression model.

The data analysis was conducted through the following procedures. First, to examine the basic information about involved variables, descriptive and correlational statistics among variables and covariates were conducted. Then, the moderated mediation analysis were examined according to the mediation analysis procedures of Hayes (2018). In detail, a series of multilevel moderated mediation models were conducted to answer our research questions with four steps. In step 1, the ICC of reading literacy was firstly calculated based on the null model to show how much the variance of reading literacy was explained by school-level factors. In step 2, a multivariable regression model (with student reading literacy as the dependent variable, three types of ICT use as independent variables, and gender and ESCS as covariates) was analyzed to show the overall associations between three types of student ICT use and reading literacy as the reference to the subsequent moderation analysis. In step 3, three moderation models (with reading literacy as dependent variable, each type of ICT use as independent variable, ICT use intensity as moderator, and the other two types of ICT use, gender and ESCS as covariates) were carried out separately to test whether student ICT use intensity moderate the association of their ICT use and reading literacy (RQ 1). In step 4, three parallel multiple moderated mediation models (with reading literacy as dependent variable, each type of ICT use as independent variable, ICT use intensity as moderator, three aspects of metacognition as parallel mediators, and the other two types of ICT use, gender and ESCS as covariates) were carried out step by step to examine the mediation of metacognition on the associations of each student ICT use and reading literacy across the four stages of ICT use intensity (RQ 2).

All analyses were conducted in *R* (version 4.2; R Core Team, 2022) for the reason that it is a free and open source software with comparative functions for statistical computing and graphics with some commercial softwares. The *PROCESS* function in *bruceR* (version 0.8.x; Bao, 2022) was used to examine the multilevel moderated mediation analysis. The *PROCESS* function can not only be used to estimate the multilevel model coefficients, but also allow for the estimations of simple slops in moderated and moderated mediation models, as well as the estimations of indirect effects in moderated mediation (Bauer and Curran, 2005; Tingley et al., 2014; Bao, 2022). In order to check for the robustness of the results, the mediation effects and standard errors were estimated based on 1000 Markov Chain Monte Carlo (Quasi-Bayesian) simulations. Missing data in continuous variables were deleted listwise, which did not change the distribution of original data (see Supplementary Table 1 for the comparisons of data before and after the listwise deletion).

## Results

To better answer the research questions in this study, we made a descriptive statistical analysis on the variables and their relationships at first (see section “Descriptive ***S***tatistics:). Then, we conducted a multivariate regression analysis to explore the overall associations of three types of ICT use with reading literacy (see section “Association of ICT Use With Reading Literacy”). To answer the first research question, we explored the moderation of ICT use intensity on the effect of ICT use on reading literacy (see section “The Moderation of ICT Use Intensity”). On this basis, we further examined the mediation of metacognition on the link between ICT use and reading literacy across ICT use intensity by conducting three multilevel moderated mediation analysis (see section “The Moderated Mediation Analysis of Metacognition Across ICT Use Intensity”), to answer the second research question.

### Descriptive Statistics

To show the summary information of involved variables, the descriptive statistics and bivariate correlations among variables are presented in Table 4. It showed that three types of student ICT use are positively correlated with each other and the correlations ranged from 0.30 to 0.50, same as the three aspects of metacognition with correlations ranged from 0.36 to 0.49. At the same time, student ICT use showed some overall significantly and negatively small links with metacognition and reading literacy with coefficients ranged from −0.01 to −0.15. Further, the three aspects of metacognition were positively linked with reading literacy, and the correlations range from 0.37 to 0.46. In addition, gender was negatively associated with three aspects of metacognition and reading literacy, indicating that boys had a better performance in metacognition and reading literacy than girls. ESCS was positively associated with metacognition and reading literacy and the correlations ranged from 0.09 to 0.21. Because results of the associations among covariates with metacognition and reading literacy were consistent with the results of subsequent moderated mediation model and our interest lies in the associations among ICT use, metacognition and reading literacy, the results of covariates with these variables were shown in Supplementary Tables 2–4 but not reported again in the subsequent model analysis.

**TABLE 4:** Descriptive statistics and correlations of variables involved in present study.

		Mean	SD	1	2	3	4	5	6	7	8
1	Gender	1.49	0.50								
2	ESCS	−0.52	1.01	−0.10							
3	ENTUSE	0.18	1.00	0.14	0.08						
4	HOMESCH	0.15	0.92	*0.01*	0.18	0.40					
5	USESCH	−0.12	1.07	0.06	0.11	0.30	0.49				
6	UR	−0.26	0.99	−0.20	0.10	−0.07	−***0.03***	−0.09			
7	SM	−0.50	0.99	−0.19	0.12	−0.08	−***0.03***	−0.08	0.49		
8	AC	−0.10	1.05	−0.12	0.09	−0.08	−0.06	−0.16	0.35	0.40	
9	READ	534.75	93.47	−0.14	0.21	−0.04	−*0.02*	−0.14	0.37	0.40	0.46

*All correlations are significant at p < 0.001 except for the italic numbers of p > 0.05 and bold italic number of p < 0.05.*

*ENTUSE, ICT use at home for recreational activities; HOMESCH, ICT use at home for schoolwork; USESCH, ICT use at school in general; UR,Understanding and Remembering (one aspect of metacognition); SM, Summarizing (one aspect of metacognition); AC, Assessing credibility of information (one aspect of metacognition); Ratio-UR, the ratio of the indirect effect from UR divided by the total effect; READ, Reading literacy.*

### Association of Information and Communication Technologies Use With Reading Literacy

Before conducting the moderated analysis, a multivariate regression including three types of ICT use and covariates as independent variables was conducted to explore the overall total effects of ICT use on reading literacy. The results (see details in Supplementary Tables 2–4) showed that student ICT use as well as covariates explained about 2% variance of their reading literacy. In terms of the coefficients, USESCH is negatively linked with their reading literacy (beta = −10.38, se = 1.22, *p* < 0.001), indicating that students with more ICT use at schools gained a lower score in reading performance. No significant associations were found between HOMESCH and ENTUSE with reading literacy.

### The Moderation of Information and Communication Technologies Use Intensity

To answer the research question 1, the ICT use intensity was added into the model to examine the moderation. The original model results were shown in Supplementary Tables 2–4 and the refined moderation results were shown in Table 5. As shown in Table 5, the corresponding ICT use intensity plays a moderating role in the associations of reading literacy with ENTUSE [F (3,5038) = 52.08, *p* < 0.001], HOMESCH [F (3,5046) = 74.28, *p* < 0.001], and SCHUSE [F (3,5037) = 9.78, *p* < 0.001], which supports H1a, H1b, and H1c.

**TABLE 5:** The moderation analysis of ICT use level.

Path	Low use	Intermediate use	Intensive use	Excessive use
ENTUSE**→** READ	33.43*** (3.09)	6.80 (21.78)	−26.07 (16.85)	−12.48*** (2.06)
HOMESCH**→** READ	36.44*** (3.15)	76.99** (24.16)	−121.01*** (15.44)	−15.00*** (2.79)
SCHUSE**→** READ	11.83 (7.04)	4.49 (9.84)	−40.54* (17.33)	−24.58*** (3.06)

*Unstandardized regression coefficients are displayed, with standard errors in parentheses.*

*ENTUSE, ICT use at home for recreational activities; HOMESCH, ICT use at home for schoolwork; USESCH, ICT use at school in general; READ, Reading literacy.*

*p < 0.05, **p < 0.01, ***p < 0.001.

Although no overall significant association was found between ENTUSE and reading literacy, the results of the moderation analysis (see Table 5 for details) showed that this association is moderated by ENTUSE intensity. Specifically, the low ENTUSE is positively linked with reading literacy (beta = 33.43, se = 3.09, *p* < 0.001), while this positive association tends to decrease, even to negative effects with the increase of ENTUSE. It showed that the association is non-significant for students with intermediate (beta = 6.80, se = 21.78, ns) and intensive (beta = −26.07, se = 16.85, ns) ENTUSE, and further had a significant negative effect for students with excessive ENTUSE (beta = −12.48, se = 2.06, *p* < 0.001). An additional 3% variance of reading literacy is accounted for by the introduction of ENTUSE intensity.

The change trend of the association of HOMESCH with reading literacy is different from with that of ENTUSE. The positive association of low HOMESCH and reading literacy (beta = 36.44, se = 3.15, *p* < 0.001) first experienced a fluctuated increase for intermediate HOMESCH (beta = 76.99, se = 24.16, *p* < 0.01) and then sharply became dramatically negative for students with intensive HOMESCH (beta = −121.01, se = 15.44, *p* < 0.001), which was followed by a relatively small but stable negative effect for the excessive HOMESCH (beta = −15.00, se = 2.79, *p* < 0.001). An additional 4% variance of reading literacy is accounted for by the introduction of HOMESCH intensity.

Different from the complex associations of HOMESCH with reading literacy across use intensity, the association between SCHUSE with reading literacy is similar to that of ENTUSE. Table 5 shows that the positive association of SCHUSE with reading literacy firstly decreases and then becomes negative. However, only the negative association of excessive SCHUSE with reading literacy is statistically significant (beta = −24.58, se = 3.06, *p* < 0.001).

### The Moderated Mediation Analysis of Metacognition Across Information and Communication Technologies Use Intensity

To answer the research question 2, three multilevel moderated mediation analysis with random intercept were conducted by considering ENTUSE, HOMESCH, and SCHUSE as predictor, respectively. The results are presented in the following section.

#### Mediation of Metacognition Across ENTUSE Intensity

First, we examined the mediation of metacognition on the link between ENTUSE and reading literacy across ENTUSE intensity. To support this analysis, the hypotheses of H2a, H3a, H4a, H5a, H6a, H7a, and H8a were examined. The results in Table 6 showed that ENTUSE intensity moderated the associations of student ENTUSE with UR [F (3,5100) = 27.79, *p* < 0.001], SM [F (3,5090) = 22.25, *p* < 0.001], and AC [F (3,5087) = 29.36, *p* < 0.001], which provided empirical evidence for H2a, H3a, and H4a. The association pattern of ENTUSE with UR, SM, and AC is similar and consistent with the association of ENTUSE with reading literacy, which begins with a significant positive association, then decreases to a non-significant positive link and further to a fluctuated negative link, finally ending with a significant negative link.

**TABLE 6:** The moderated mediation analysis across ENTUSE intensity.

	Low use	Intermediate use	Intensive use	Excessive use
**Path analysis**				
ENTUSE**→**UR	0.26*** (0.04)	0.12 (0.26)	−0.39 (0.20)	−0.14*** (0.02)
ENTUSE**→**SM	0.23*** (0.04)	0.27 (0.26)	−0.37 (0.20)	−0.13*** (0.02)
ENTUSE**→**AC	0.29*** (0.04)	0.03 (0.28)	−0.09 (0.22)	−0.15*** (0.03)
UR**→** READ	9.01*** (2.42)	12.36*** (2.35)	9.53*** (2.29)	11.30*** (2.34)
SM**→** READ	13.02*** (2.37)	11.39*** (2.43)	13.95*** (2.32)	15.11*** (2.38)
AC**→** READ	23.76*** (2.14)	20.38*** (2.09)	21.64*** (2.03)	18.98*** (2.15)
ENTUSE**→** READ	22.31*** (2.94)	3.27 (19.88)	−15.42 (15.40)	−6.62*** (1.94)
**Mediation analysis**				
ENTUSE**→**UR**→** READ	2.34** (0.73)	1.61 (3.20)	−3.79 (2.22)	−1.56*** (0.43)
ENTUSE**→**SM**→** READ	2.94*** (0.74)	3.19 (3.06)	−5.14 (3.05)	−1.93*** (0.48)
ENTUSE**→**AC**→** READ	6.95*** (1.15)	0.81 (5.55)	−1.92 (4.74)	−2.85*** (0.60)
Total effect	33.43***	6.80	−26.07	−12.48***
Total indirect effect	12.23			−6.34
Ratio-UR	7.00%			12.50%
Ratio-SM	8.79%			15.46%
Ratio-AC	20.79%			22.84%
Ratio-total indirect	36.58%			50.80%

*Unstandardized regression coefficients are displayed, with standard errors in parentheses. ENTUSE, ICT use at home for recreational activities; HOMESCH, ICT use at home for schoolwork; USESCH, ICT use at school in general; READ, Reading literacy; UR, Understanding and Remembering (one aspect of metacognition); SM, Summarizing (one aspect of metacognition); AC, Assessing credibility of information (one aspect of metacognition); Ratio-UR, the ratio of the indirect effect from UR divided by the total effect; Ratio-SM, the ratio of the indirect effect from SM divided by the total effect; Ratio-AC, the ratio of the indirect effect from AC divided by the total effect; Ratio-Total indirect, the ratio of total indirect effect from metacognition (UR+SM+AC) divided by the total effect.*

*p < 0.05, **p < 0.01, ***p < 0.001.

Second, the links of metacognition with reading literacy were examined. It was found that student ENTUSE intensity did not moderate the link of student reading literacy with UR, SM, and AC, which did not support H5a, H6a, and H7a. The path coefficients of each metacognition component with reading literacy across four ENTUSE intensity stages were comparable and significant (see details in Table 6). Another finding was that AC showed an overall more important role in reading literacy with higher coefficients than SM, followed by UR.

Third, the moderation of ENTUSE intensity was explored further after introducing metacognition into this model. The results indicated that ENTUSE intensity still moderated the link of student reading literacy with ENTUSE [F (3,5026) = 23.17, *p* < 0.001] after considering the influence of mediators, supporting H8a. Compared with the direct link in the simple moderation model, all corresponding links of ENTUSE with reading literacy across four intensity stages decreased. In this case, only the positive link of low ENTUSE (beta = 22.31, se = 2.94, *p* < 0.001) and negative link of excessive ENTUSE (beta = −6.62, se = 1.94, *p* < 0.001) on reading literacy were significant.

The results showed that UR, SM, and AC mediated the link of ENTUSE with reading literacy across ICT use intensity. The AC mediated the most effect, followed by SM and UR in both of low and excessive ENTUSE. In detail, for student group with low ENTUSE, the mediation amount of UR was 2.34 (0.26 × 9.01 = 2.34, se = 0.73, *p* < 0.05), which accounted for 7.00% of total effects that ENTUSE showed on reading literacy. And the corresponding mediation amount of SM and AC was 2.94 (8.79%) and 6.95 (20.79%), respectively. A total of 36.58% of the total effect that ENTUSE showed on reading literacy was accounted for by three aspects of metacognition for low ENTUSE. The mediation amount of UR, SM, and AC was −1.56 (12.50%), −1.93 (15.46%), and −2.85 (22.84%), which combined accounted for 50.80% of the total effect for excessive ENTUSE. Thus, metacognition demonstrated a more important role in mediating the effects of excessive rather than low ENTUSE.

#### Mediation of Metacognition Across HOMESCH Intensity

The results in Table 7 show that HOMESCH intensity moderates the links of student HOMESCH with UR [F (3,5125) = 60.35, *p* < 0.001], SM [F (3,5113) = 62.95, *p* < 0.001], and AC [F (3,5108) = 78.56, *p* < 0.001], which supports H2b, H3b, and H4b. The link pattern of HOMESCH with UR, SM, and AC across four HOMESCH intensity stages is similar and consistent with the link of HOMESCH with reading literacy, which begins with a significant positive link of low HOMESCH, then turns to a more pronounced significant effect of intermediate HOMESCH, followed by a pronounced negative effect of intensive HOMESCH, and finally ends with a stable significant negative link of excessive HOMESCH.

**TABLE 7:** The moderated mediation analysis across HOMESCH intensity.

	Low use	Intermediate use	Intensive use	Excessive use
**Path analysis**				
HOMESCH**→**UR	0.36*** (0.04)	0.58* (0.29)	−1.42*** (0.19)	−0.19*** (0.03)
HOMESCH**→**SM	0.33*** (0.04)	0.68* (0.29)	−1.76*** (0.19)	−0.15*** (0.03)
HOMESCH**→**AC	0.42*** (0.04)	0.82** (0.31)	−1.86*** (0.20)	−0.21*** (0.04)
UR**→** READ	9.09*** (2.41)	8.63*** (2.34)	12.60*** (2.17)	11.03*** (2.52)
SM**→** READ	9.52*** (2.44)	14.56*** (2.32)	12.23*** (2.23)	16.38*** (2.57)
AC**→** READ	18.46*** (2.14)	22.49*** (2.09)	20.14*** (1.94)	22.62*** (2.33)
HOMESCH**→** READ	24.14*** (3.11)	46.25* (22.24)	−48.85** (15.03)	−5.79* (2.65)
**Mediation analysis**				
HOMESCH**→**UR**→** READ	3.27*** (0.95)	5.07 (2.87)	−18.00*** (3.91)	−2.05*** (0.60)
HOMESCH**→**SM**→** READ	3.17*** (0.91)	10.00* (4.45)	−21.43*** (4.58)	−2.51*** (0.67)
HOMESCH**→**AC**→** READ	7.68*** (1.17)	18.68** (7.01)	−37.50*** (5.40)	−4.71*** (0.95)
Total effect	36.44***	76.99**	−121.01***	−15.00***
Total indirect	14.12	28.68	−76.93	−9.27
Ratio-UR	8.97%	–	14.87%	13.67%
Ratio-SM	8.70%	12.99%	17.71%	16.73%
Ratio-AC	21.08%	24.26%	30.99%	31.40%
Ratio- Total indirect	38.75%	37.25%	63.57%	61.80%

*Unstandardized regression coefficients are displayed, with standard errors in parentheses.*

*ENTUSE, ICT use at home for recreational activities; HOMESCH, ICT use at home for schoolwork; USESCH, ICT use at school in general; READ, Reading literacy; UR, Understanding and Remembering (one aspect of metacognition); SM, Summarizing (one aspect of metacognition); AC, Assessing credibility of information (one aspect of metacognition); Ratio-UR, the ratio of the indirect effect from UR divided by the total effect; Ratio-SM, the ratio of the indirect effect from SM divided by the total effect; Ratio-AC, the ratio of the indirect effect from AC divided by the total effect; Ratio-Total indirect, the ratio of total indirect effect from metacognition (UR+SM+AC) divided by the total effect.*

*p < 0.05, **p < 0.01, ***p < 0.001.

Regarding the links of metacognition with reading literacy, it was found that AC showed a stronger link than SM and UR. Further, there is no difference among the positive and significant links of each metacognition with reading literacy across four stages of ICT use intensity, which does not support H5b, H6b, and H7b. The path coefficients of each metacognition with reading literacy across four HOMESCH intensity stages are shown in Table 7.

In addition, there is a sharp decrease in the direct association of HOMESCH with reading literacy across four HOMESCH intensity stages after the introduction of metacognition into the model, but all links are still significant. The differences between these links across four stages are still significant, which means that HOMESCH intensity still moderates the link of student reading literacy with HOMESCH [F (3,5026) = 23.17, *p* < 0.001] after considering the influence of mediators, providing support for H8b.

In terms of the mediation amount, the results show that UR, SM, and AC mediate the link of HOMESCH with reading literacy for students in all four stages except for the UR for intermediate HOMESCH. The AC mediates the most effects, followed by SM, and UR. A total of 61.8% negative effects of intensive HOMESCH and 63.57% negative effect of excessive HOMESCH on reading literacy is accounted for by metacognition, while only about 38.75% positive effects of low HOMESCH and 37.25% positive effects of intermediate HOMESCH are mediated.

#### Mediation of Metacognition Across USESCH Intensity

The results in Table 8 show that the link of student USESCH with UR [F (3,5101) = 7.01, *p* < 0.001], SM [F (3,5089) = 9.89, *p* < 0.001], and AC [F (3,5087) = 13.85, *p* < 0.001] are moderated by USESCH intensity, supporting H2c, H3c, and H4c. To be more precise, there is a fluctuated and pronounced negative effect of intensive USESCH (beta = −0.62, se = 0.21, *p* < 0.01) and a stable but smaller negative effect of excessive USESCH (beta = −0.24, se = 0.04, *p* < 0.001) on UR. The link of USESCH with SM and AC is similar and both show a positive effect of low USESCH (SM/AC: beta = 0.29/0.30, se = 0.08/0.09, *p* < 0.001) and a negative effect of excessive USESCH (SM/AC: beta = −0.20/−0.29, se = 0.04/0.04, *p* < 0.001).

**TABLE 8:** The moderated mediation analysis across USESCH intensity.

	Low use	Intermediate use	Intensive use	Excessive use
**Path analysis**				
USESCH**→**UR	0.10 (0.08)	0.04 (0.12)	−0.62** (0.21)	−0.24*** (0.04)
USESCH**→**SM	0.29*** (0.08)	−0.07 (0.12)	−0.40 (0.21)	−0.20*** (0.04)
USESCH**→**AC	0.30*** (0.09)	0.06 (0.12)	−0.37 (0.22)	−0.29*** (0.04)
UR**→** READ	9.09*** (2.31)	10.12*** (2.43)	11.44*** (2.36)	13.44*** (2.35)
SM**→** READ	16.63*** (2.37)	12.38*** (2.40)	11.94*** (2.36)	12.94*** (2.44)
AC**→** READ	19.34*** (2.09)	22.06*** (2.10)	22.08*** (2.12)	21.91*** (2.20)
USESCH**→** READ	0.51 (6.46)	3.50 (8.97)	−20.86 (15.84)	−13.51 (2.88)***
**Mediation amount**				
USESCH**→**UR**→** READ	0.93 (0.83)	0.42 (1.19)	−7.18* (2.88)	−3.14*** (0.74)
USESCH**→**SM**→** READ	4.86** (1.58)	−0.84 (1.47)	−4.76 (2.75)	−2.53*** (0.66)
USESCH**→**AC**→** READ	5.94** (1.89)	1.46 (2.70)	−8.27 (4.99)	−6.25*** (1.07)
Total indirect	10.8	–	−7.18*	11.92***
Total effect	11.83	4.49	−40.54	−24.58
Ratio-UR	–	–	17.71%	12.77%
Ratio-SM	41.08%	–	–	10.29%
Ratio-AC	50.21%	–	–	25.43%
Ratio-total indirect	91.29%	–	17.71%	48.49%

*Unstandardized regression coefficients are displayed, with standard errors in parentheses.*

*ENTUSE, ICT use at home for recreational activities; HOMESCH, ICT use at home for schoolwork; USESCH, ICT use at school in general; READ, Reading literacy; UR, Understanding and Remembering (one aspect of metacognition); SM, Summarizing (one aspect of metacognition); AC, Assessing credibility of information (one aspect of metacognition); Ratio-UR, the ratio of the indirect effect from UR divided by the total effect; Ratio-SM, the ratio of the indirect effect from SM divided by the total effect; Ratio-AC, the ratio of the indirect effect from AC divided by the total effect; Ratio-Total indirect, the ratio of total indirect effect from metacognition (UR+SM+AC) divided by the total effect.*

*p < 0.05, **p < 0.01, ***p < 0.001.

Regarding the links of metacognition with reading literacy, similar with that of HOMESCH, it was found that AC showed a stronger link than SM and UR. There is no difference among the positive and significant links of each metacognition with reading literacy across USESCH intensity, which does not support H5c, H6c, and H7c. The path coefficients of each metacognition with reading literacy across four stages of USESCH intensity are shown in Table 8.

SCHUSE intensity does not moderate the link of student reading literacy with SCHUSE, with no support for H8c. The results show that only the negative effects of excessive USESCH on reading literacy is statistically significant (beta = −13.51, se = 2.88, *p* < 0.001).

In terms of the mediation amount, the results show that the positive non-significant total effect of low USESCH (beta = 11.83) is almost fully mediated by SM and AC [SM: 4.86 (se = 1.58, *p* < 0.01, ratio = 41.08%]; AC: 5.94 (se = 1.89, *p* < 0.01, ratio = 50.21%). Approximately 17.71% (beta = −7.18, se = 2.88, *p* < 0.01) of the total negative effect of intensive USESCH is mediated by UR. A total of 48.49% total negative effect of excessive USESCH is mediated by UR (beta = −3.14, se = 0.74, *p* < 0.001, ratio = 12.77%), SM (beta = −2.53, se = 0.66, *p* < 0.001, ratio = 10.29%), and AC (beta = −6.25, se = 1.07, *p* < 0.001, ratio = 25.43%).

### The Summary of Hypotheses Test

In summary, the results of present study show that ICT use intensity not only moderates the links between three types of ICT use and reading literacy, but also moderates the mediations of metacognition on the said links. The test results of our hypotheses are summarized in Table 9.

**TABLE 9:** The summary of our hypotheses test.

	Supported	Not supported	Content of hypotheses
H1	H1a, H1b, H1c		ICT use intensity moderated the link of ICT use with reading literacy
H2	H2a, H2b, H2c		ICT use intensity moderated the link of ICT use with UR.
H3	H3a, H3b, H3c		ICT use intensity moderated the link of ICT use with SM.
H4	H4a, H4b, H4c		ICT use intensity moderated the link of ICT use with AC.
H5		H5a, H5b, H5c	ICT use intensity did not moderate the link of UR with reading literacy.
H6		H6a, H6b, H6c	ICT use intensity did not moderate the link of SM with reading literacy.
H7		H7a, H7b, H7c	ICT use intensity did not moderate the link of AC with reading literacy.
H8	H8a, H8b	H8c	ENTUSE and HOMESCH intensity moderated the link of ENTUSE and HOMESCH with reading literacy, while USESCH intensity did not moderate the corresponding link, after controlling for the mediation of metacognition.

*ENTUSE, ICT use at home for recreational activities; HOMESCH, ICT use at home for schoolwork; USESCH, ICT use at school in general; UR, Understanding and Remembering (one aspect of metacognition); SM, Summarizing (one aspect of metacognition); AC, Assessing credibility of information (one aspect of metacognition).*

## Discussion

A group of studies have explored the links of student ICT use and reading literacy (Hu et al., 2018; Gubbels et al., 2020; Vazquez-Lopez and Huerta-Manzanilla, 2021; Kong et al., 2022), and mixed results were found across studies. However, those studies failed to explain the mechanisms that resulted in the ambiguous findings. The present study explored the specific links of three types of student ICT use and reading literacy across ICT use intensity as well as the mediation of metacognition on this link across ICT use intensity. We found a similar effect pattern of ICT use on reading literacy with the increase in ICT use intensity across three types of ICT use. In addition, metacognition positively mediated the link of lower ICT use (e.g., low ENTUSE, low and intermediate HOMESCH) with reading literacy, and negatively mediated the link of intensive and excessive ICT use with reading literacy. This study extends previous understanding of the relationship between student ICT use and reading literacy, and can provide insightful information to the implementation of educational decisions or interventions to administer ICT tools to facilitate student reading literacy and learning process.

### The Moderation of Information and Communication Technologies Use Intensity

The findings of this study confirmed our hypothesis that student ICT use intensity moderated the links of all three types of ICT use and their reading literacy, which is consistent with the research of (Gubbels et al., 2020). Student ICT use intensity was classified into four groups: low use, intermediate use, intensive use, and excessive use according to the cutoff values in the 25, 50, and 70% percentile of student ICT use. With the increase in ICT use intensity, it begins with a sound positive effect of low ICT use on reading literacy, which further experiences a fluctuated decrease to a smaller significant for intermediate use, and further turns to a fluctuated negative effect for intensive use, followed by a more stable but smaller negative effect of excessive ICT use.

This similar effect pattern across ICT use intensity can be explained as follows. The positive effects of low ICT use on reading literacy can be explained by the fact that the original introduction of the computer and broadband enabled student access to more useful resources and provided helpful and interesting functions that attracted students’ attention to learning (Barrow et al., 2009; Belo et al., 2016). With the increasing intensity of ICT use, and as ICT tools were expanded to be used in wider areas and in diverse ways, such as for leisure or social activities, student attention begins to be distracted from learning to some degree (Leuven et al., 2007). Thus, the effects of intermediate ICT use may decrease and show an overall less positive or even non-significant effect. If these issues are not addressed, the less positive or non-significant effects may further turn to negative for intensive use because students may spend too much time on ICT, and little time will be left for their learning and development of other related constructs, such as creativity and critical thinking. This can be supported by the serious implications of the problematic use of the Internet and ICT tools (Mamun et al., 2020). However, if students with intensive ICT use can cope with the side effects of ICT use in an appropriate manner, such as seeking the help of their parents or teachers, the negative effects may be reduced and the increasing familiarity with ICT tools would even enhance their reading. But these students are proportionately small in number. Thus, overall negative but fluctuated effects were found for intensive ICT use on reading literacy. After the fluctuation, stable damaging effects of excessive ICT use on reading literacy were found as excessive use would hinder student cognitive process and critical thinking by increasing working and cognitive load (Lorenzo and Trujillo, 2018).

The magnitude and dominants of these effects vary across ICT use intensity stages and use purpose, which leads to the diverse overall effects resulting from the compensation of effects across four intensity stages and use types. This can be confirmed by our finding that student ICT use at home both for leisure activities and schoolwork showed overall null effects on their reading literacy, while the ICT use at school in general showed an overall sound negative effect on their reading literacy. Thus, although null effects were found in ICT use at home both for leisure activities and schoolwork, the specific magnitude and dominant stages of different ICT use intensity stage are different across these two types of ICT use. In terms of the link of ICT use at home for leisure work, the positive link of low ENTUSE and the stable negative link of excessive ENTUSE are statistically significant and they compensated each other, which lead to the overall null effect, while for ICT use at home for schoolwork, all the links of four ICT use intensity are statistically significant. The positive effect of intermediate HOMESCH as well as the negative effect of intensive HOMESCH dominates the overall effect with a sound larger effect than the other two stages. The positive effects of the former two stages compensated the effects of the intensive stages, which results in the null overall effects. Regarding the links of ICT use at school, the excessive USESCH dominates the whole work, resulting in an overall negative effect.

### Moderated Mediation of Metacognition Across Information and Communication Technologies Use Intensity

To further explore the mechanism of how ICT use functions in regards to reading literacy, this study explored the mediation of metacognition. Consistent with the study of Lee and Wu (2013), which showed that metacognition mediated the effect on student social media use (i.e., ICT use for entertainment) and reading literacy, this study extended this line of inquiry and found that all of three aspects of metacognition (i.e., understanding and remembering, summarizing, and assessing credibility) mediated the effects of the three types of ICT use on reading literacy. Further, we explored the moderated mediation across four ICT use intensity stages. The results showed that all three aspects of metacognition can mediate the positive effects of low and intermediate ICT use both at home and at school on reading literacy, and the negative effects of intensive and excessive ICT use. This indicated that appropriate ICT use benefits student metacognition, while intensive or excessive ICT use undermines student metacognition, which in turn reduced student reading literacy. Taking the undermining effect on assessing credibility as an example, in the intensive or excessive ICT use scenario, students may read the same materials posted in different sites or by different individuals, and they may easily misjudge them as credible (Macedo-Rouet et al., 2020). Thus, developing student metacognition, especially for students with intensive or excessive ICT use is an effective way to reduce the side effect of ICT use on reading literacy.

The development of student metacognition can start from improving student ability in assessing the quality and credibility of information. The present study also found that assessing credibility of information showed a more pronounced mediation than summarizing, followed by understanding and remembering. This larger mediation is mainly from the larger effects of assessing credibility on reading literacy rather than the link with ICT use, compared to the other two aspects. Since the digital era is characterized by massive information flows, students need to be able to distinguish between fact and opinion, and detect biased information and malicious content such as phishing emails or fake news (Suarez-Alvarez, 2021). This study also confirmed the growing importance of student ability in assessing the credibility of information (OECD, 2019).

### Limitation

There were some limitations to this study. First, regarding the cross-sectional data in this research, it is confined to making directional assumptions about the links between ICT use, metacognition, and reading literacy, but not a causal relationship. However, due to the large sample size employed by the study, the results are still valuable despite the cross-sectional design. Longitudinal research can be adopted in the future to test the causal links of the above variables. Second, all the measures except for that of reading literacy were based on self-reportage, which may cast some doubts on their validity due to respondents’ social desirability response bias. In addition, only the frequency of ICT use was measured. Therefore, other measures on more diverse aspects of ICT use, such as the duration of use per login and the length of users’ memberships for the actual use records, may better explore the link between ICT use and reading literacy. Third, to simplify the complex analysis, the results of present study were reported based on the analysis with one random plausive value of student reading literacy, which is a small methodological flaw of present study. Although there’s no substantial difference for using one random plausive value and several plausive values on large sample, the aggregated results for several plausive values are incontestable from a theoretical point of view (OECD, 2009, p. 46). Thus, future study could use more plausive values instead of using one random plausive value to have a more robust result. Finally, only 15-year-olds from Hong Kong were examined in this research, which needs to be replicated by other samples from different cultural backgrounds to generalize the findings.

## Conclusion

Present study found a dynamic effect pattern for the link between ICT use and reading literacy with the increasing intensity of ICT use, which may account for the existing inconsistent results. The overall effect pattern can be described as below: it begins with a positive effect of low ICT use, then experiences a fluctuated decrease to a small but still positive effect of intermediate use, and further turns to a fluctuated negative effect for intensive use, and finally ends up with a stable negative effect of excessive use. In addition, we found that all three aspects of metacognition play an important role in accounting for the positive effects of low or intermediate ICT use and negative effects of intensive and excessive use on reading literacy.

Therefore, we recommend that researchers and teachers design more metacognitive scaffolds that can help learners manage their cognitive processing, direct their actions, off-load their working memory, to alleviate the side effects of intensive or excessive ICT use on reading literacy. In the aspect of scaffolds for understanding and remembering, we suggest providing more question and answer spaces in ICT-based learning environment to guide students reading with a problem-solving perspective, including predicting expectations as text unfolds, connecting content as reading proceeds, retrieving information to seeking and building meaning (Beck and McKeown, 2006; McKeown and Beck, 2009). In terms of scaffolds for summarizing, we suggest creating more summary framework or software to help students select main information, delete trivial information, and relate to supporting information during reading. As for scaffolds for assessing credibility, we suggest popularizing the methods and techniques of differentiating information to students, so as to assist them in distinguishing false information from true information. In addition, we recommend that developers of reading program develop more ICT-assisted cognitive tools for understanding, summarizing, and assessing information, such as XMind for visualizing text ideas, Evernote for making annotations, and reflection tools for self-assessment.

## Data Availability Statement

Publicly available datasets were analyzed in this study. This data can be found here: Student questionnaire data file at https://www.oecd.org/pisa/data/2018database/.

## Ethics Statement

The studies involving human participants were reviewed and approved by Organisation for Economic Co-operation and Development (OECD). Written informed consent to participate in this study was provided by the participants or their legal guardian/next of kin.

## Author Contributions

ML contributed to the conception of the study, the data analysis, manuscript preparation, and wrote the manuscript. MW helped perform the analysis with constructive discussions and wrote the manuscript. Both authors contributed to the article and approved the submitted version.

## Conflict of Interest

The authors declare that the research was conducted in the absence of any commercial or financial relationships that could be construed as a potential conflict of interest.

## Publisher’s Note

All claims expressed in this article are solely those of the authors and do not necessarily represent those of their affiliated organizations, or those of the publisher, the editors and the reviewers. Any product that may be evaluated in this article, or claim that may be made by its manufacturer, is not guaranteed or endorsed by the publisher.

## Footnotes

1
https://www.oecd.org/pisa/data/2018database/
