# Supplementary material for: Examining the Relationship of Information and Communication Technologies Use and Reading Literacy: A Moderated-Mediation Analysis of Metacognition Across Information and Communication Technologies Use Intensity
Source: Front Psychol. 2022 Jul 6;13:916497. doi: 10.3389/fpsyg.2022.916497 (PMC9297003; doi:10.3389/fpsyg.2022.916497)
Supplement: Supplementary file 1 [file Data_Sheet_1.PDF]

Supplementary Material

1 Supplementary Tables

Table 1 The comparisons of variables before and after the listwise deletion

	Original data<br>Mean (SD)	Complete data<br>Mean (SD)	t	Difference [95% CI]
Gender	1.49 (0.50)	1.50 (0.50)	0.40	0.00[–0.02, 0.02]
ESCS	-0.52 (1.01)	-0.52 (1.01)	0.07	0.00[–0.04, 0.04]
ENTUSE:	0.18 (1.00)	0.18 (1.01)	-0.24	–0.00[–0.04, 0.03]
HOMESCH:	0.15 (0.92)	0.15 (0.93)	-0.17	–0.00[–0.04, 0.03]
USESCH	-0.12 (1.07)	-0.12 (1.07)	0.06	0.00[–0.04, 0.04]
UR	-0.26 (0.99)	-0.27 (0.99)	-0.57	–0.01[–0.05, 0.03]
SM	-0.50 (0.99)	-0.51 (0.99)	-0.57	–0.01[–0.05, 0.03]
AC	-0.10 (1.05)	-0.11 (1.05)	-0.83	–0.02[–0.06, 0.02]
READ:	534.75 (93.47)	531.34 (95.12)	-1.87	–3.41[–6.98, 0.16]

Note: Mean values are displayed with standard errors in parentheses for the first two columns. There are no any significant differences for all variables before and after the missing values were deleted listwise because all p-values are larger than 0.05.

Table 2 The path analysis of moderated mediation across ENTUSE intensity

	(0) READ	(1) READ	(2) UR	(3) SM	(4) AC	(5) READ
(Intercept)	530.48***	553.90***	–0.08	–0.33***	0.11*	546.28***
	(4.21)	(5.30)	(0.05)	(0.05)	(0.05)	(4.55)
Gender	–11.75***	–8.97***	–0.29***	–0.28***	–0.14***	–0.30
	(2.45)	(2.42)	(0.03)	(0.03)	(0.03)	(2.23)
ESCS	3.67**	3.55**	0.04**	0.04**	0.02	3.52**
	(1.29)	(1.26)	(0.01)	(0.01)	(0.02)	(1.15)
HOMESCH	–1.63	–0.91	0.01	–0.00	0.01	–0.89
	(1.48)	(1.46)	(0.02)	(0.02)	(0.02)	(1.33)
USESCH	–10.38***	–9.64***	–0.06***	–0.05***	–0.12***	–6.17***
	(1.22)	(1.20)	(0.01)	(0.01)	(0.02)	(1.10)
ENTUSE	1.05	33.43***	0.26***	0.23***	0.29***	22.31***
	(1.23)	(3.09)	(0.04)	(0.04)	(0.04)	(2.94)
ENTUSE2		–13.98	–0.08	–0.02	–0.13	–9.56
		(7.40)	(0.09)	(0.09)	(0.09)	(6.81)
ENTUSE3		–13.06**	–0.09	–0.10	–0.13*	–8.22
		(4.51)	(0.05)	(0.05)	(0.06)	(4.20)
ENTUSE4		–15.29**	–0.16**	–0.16**	–0.18**	–8.14
			(0.06)	(0.06)	(0.06)	(4.47)
ENTUSE×ENTUSE2			–0.14	0.05	–0.26	–19.03
			(0.27)	(0.27)	(0.28)	(20.07)
ENTUSE×ENTUSE3			–0.65**	–0.59**	–0.38	–37.73*
			(0.21)	(0.21)	(0.22)	(15.66)
ENTUSE×ENTUSE4			–0.40***	–0.35***	–0.44***	–28.93***
			(0.04)	(0.04)	(0.05)	(3.50)
UR						9.01***
						(2.42)
SM						13.02***
						(2.37)
AC						23.76***
						(2.14)
ENTUSE2×UR						3.35
						(3.37)
ENTUSE3×UR						0.52
						(3.32)
ENTUSE4×UR						2.29
						(3.35)
ENTUSE2×SM						–1.62
						(3.38)
ENTUSE3×SM						0.93

	(0) READ	(1) READ	(2) UR	(3) SM	(4) AC	(5) READ
						(3.31)
ENTUSE4×SM						2.09
						(3.35)
ENTUSE2×AC						–3.39
						(2.97)
ENTUSE3×AC						–2.12
						(2.93)
ENTUSE4×AC						–4.79
						(3.01)
Marginal R^2	0.02	0.05	0.06	0.05	0.05	0.22
Conditional R^2	0.31	0.31	0.11	0.12	0.12	0.39
AIC	60168.19	59951.72	14095.03	14146.21	14742.33	58934.51
BIC	60220.61	60043.46	14186.77	14237.95	14834.07	59104.88
N (students)	5180	5180	5180	5180	5180	5180
N (schools)	152	152	152	152	152	152
Between variance	2479.38	2214.28	0.05	0.06	0.08	1357.24
Within variance	6010.78	5806.07	0.85	0.86	0.96	4833.86

Note. Unstandardized regression coefficients are displayed, with standard errors in parentheses. ENTUSE = ICT use at home for entertainment activities; ENTUSE2 = intermediated ENTUSE (the second classification of ENTUSE intensity, ENTUSE 1=low ENTUSE as the reference group); ENTUSE3 = intensive ENTUSE; ENTUSE4 = excessive ENTUSE;HOMESCH = ICT use at home for schoolwork; USESCH = ICT use at school in general; READ = Reading literacy; UR = Understanding and Remembering (one aspect of metacognition); SM = Summarizing (one aspect of metacognition); AC = Assessing credibility of information (one aspect of metacognition).

* $p < .05$. ** $p < .01$. *** $p < .001$.

Table 3 The path analysis of moderated mediation across HOMESCH intensity

	(0) READ	(1) READ	(2) UR	(3) SM	(4) AC	(5) READ
(Intercept)	530.48***	571.10***	0.03	–0.19***	0.27***	560.44***
	(4.21)	(5.44)	(0.05)	(0.05)	(0.05)	(4.79)
Gender	–11.75***	–7.34**	–0.27***	–0.25***	–0.10***	0.28
	(2.45)	(2.41)	(0.03)	(0.03)	(0.03)	(2.23)
ESCS	3.67**	3.94**	0.04**	0.05**	0.03	3.87***
	(1.29)	(1.26)	(0.01)	(0.01)	(0.02)	(1.15)
USESCH	–10.38***	–9.03***	–0.05***	–0.04**	–0.11***	–5.73***
	(1.22)	(1.20)	(0.01)	(0.01)	(0.02)	(1.11)
ENTUSE	1.05	3.65**	0.00	–0.01	0.00	3.82***
	(1.23)	(1.22)	(0.01)	(0.01)	(0.02)	(1.12)
HOMESCH	–1.63	36.44***	0.36***	0.33***	0.42***	24.14***
	(1.48)	(3.15)	(0.04)	(0.04)	(0.04)	(3.11)
HOME2		–19.61**	–0.09	–0.08	–0.13	–18.37**
		(6.38)	(0.08)	(0.08)	(0.08)	(6.00)
HOME3		–11.08*	0.03	0.06	0.05	–15.71**
		(5.41)	(0.06)	(0.07)	(0.07)	(5.22)
HOME4		–38.33***	–0.21**	–0.27***	–0.27***	–30.58***
		(5.43)	(0.07)	(0.07)	(0.07)	(5.11)
HOMESCH×HOME2		40.55	0.22	0.35	0.40	22.11
		(24.32)	(0.29)	(0.29)	(0.31)	(22.43)
HOMESCH×HOME3		–157.44***	–1.78***	–2.09***	–2.28***	–72.99***
		(15.80)	(0.19)	(0.19)	(0.20)	(15.35)
HOMESCH×HOME4		–51.43***	–0.55***	–0.49***	–0.63***	–29.93***
			(0.05)	(0.05)	(0.05)	(4.03)
UR						9.09***
						(2.41)
SM						9.52***
						(2.44)
AC						18.46***
						(2.14)
HOME2×UR						–0.46
						(3.35)
HOME3×UR						3.51
						(3.24)
HOME4×UR						1.94
						(3.49)
HOME2×SM						5.04
						(3.36)

	(0) READ	(1) READ	(2) UR	(3) SM	(4) AC	(5) READ
HOME3×SM						2.71
						(3.30)
HOME4×SM						6.87
						(3.54)
HOME2×AC						4.03
						(2.98)
HOME3×AC						1.68
						(2.88)
HOME4×AC						4.16
						(3.15)
Marginal R^2	0.02	0.06	0.08	0.08	0.08	0.22
Conditional R^2	0.31	0.32	0.12	0.13	0.14	0.40
AIC	60168.19	59887.68	13997.40	14026.91	14609.61	58924.64
BIC	60220.61	59979.41	14089.13	14118.64	14701.35	59095.00
N (students)	5180	5180	5180	5180	5180	5180
N (schools)	152	152	152	152	152	152
Between variance	2479.38	2199.66	0.04	0.05	0.07	1397.04
Within variance	6010.78	5734.31	0.84	0.84	0.93	4821.45

Note. Unstandardized regression coefficients are displayed, with standard errors in parentheses. ENTUSE = ICT use at home for entertainment activities; HOMESCH = ICT use at home for schoolwork; HOME2 = intermediated HOMESCH (the second classification of HOMESCH intensity, HOME1=low HOMESCH as the reference group); HOME3 = intensive HOMESCH; HOME4 = excessive HOMESCH; USESCH = ICT use at school in general; READ=reading literacy; UR = Understanding and Remembering (one aspect of metacognition); SM = Summarizing (one aspect of metacognition); AC = Assessing credibility of information (one aspect of metacognition).

* $p < .05$. ** $p < .01$. *** $p < .001$.

Table 4 The path analysis of moderated mediation across USESCH intensity

	(0) READ	(1) READ	(2) UR	(3) SM	(4) AC	(5) READ
(Intercept)	530.48***	553.33***	–0.13	–0.10	0.39**	537.33***
	(4.21)	(11.10)	(0.13)	(0.13)	(0.13)	(10.01)
Gender	–11.75***	–8.77***	–0.29***	–0.27***	–0.12***	–0.11
	(2.45)	(2.43)	(0.03)	(0.03)	(0.03)	(2.24)
ESCS	3.67**	3.96**	0.04**	0.05**	0.03*	3.77**
	(1.29)	(1.27)	(0.01)	(0.01)	(0.02)	(1.15)
HOMESCH	–1.63	1.78	0.04*	0.03	0.05**	0.50
	(1.48)	(1.49)	(0.02)	(0.02)	(0.02)	(1.36)
ENTUSE	1.05	3.27**	–0.00	–0.02	–0.00	3.50**
	(1.23)	(1.24)	(0.01)	(0.01)	(0.02)	(1.13)
USESCH	–10.38***	11.83	0.10	0.29***	0.30***	0.51
	(1.22)	(7.04)	(0.08)	(0.08)	(0.09)	(6.46)
USESCH2		–4.48	–0.02	–0.25	–0.24	4.35
		(10.72)	(0.13)	(0.13)	(0.14)	(9.86)
USESCH3		–4.16	0.17	–0.23	–0.34*	4.07
		(12.27)	(0.15)	(0.15)	(0.16)	(11.26)
USESCH4		–19.06	–0.07	–0.37**	–0.49***	–3.22
		(11.18)	(0.13)	(0.13)	(0.14)	(10.25)
USESCH×USESCH2			–0.06	–0.36*	–0.24	2.99
			(0.15)	(0.15)	(0.15)	(11.04)
USESCH×USESCH3			–0.72**	–0.69**	–0.68**	–21.37
			(0.22)	(0.22)	(0.24)	(17.12)
USESCH×USESCH4			–0.34***	–0.49***	–0.59***	–14.02*
			(0.09)	(0.09)	(0.10)	(7.09)
UR						9.09***
						(2.31)
SM						16.63***
						(2.37)
AC						19.34***
						(2.09)
USESCH2×UR						1.03
						(3.35)
USESCH3×UR						2.34
						(3.30)
USESCH4×UR						4.35
						(3.29)
USESCH2×SM						–4.25
						(3.37)
USESCH3×SM						–4.69

	(0) READ	(1) READ	(2) UR	(3) SM	(4) AC	(5) READ
						(3.34)
USESCH4×SM						–3.69
						(3.40)
USESCH2×AC						2.72
						(2.95)
USESCH3×AC						2.74
						(2.97)
USESCH4×AC						2.57
						(3.02)
Marginal R^2	0.02	0.04	0.06	0.06	0.06	0.21
Conditional R^2	0.31	0.31	0.11	0.12	0.13	0.39
AIC	60168.19	60008.96	14107.63	14135.91	14702.39	58980.32
BIC	60220.61	60100.69	14199.37	14227.65	14794.12	59150.69
N (students)	5180	5180	5180	5180	5180	5180
N (schools)	152	152	152	152	152	152
Between variance	2479.38	2319.76	0.05	0.07	0.08	1411.62
Within variance	6010.78	5866.22	0.85	0.85	0.95	4874.15

Note. Unstandardized regression coefficients are displayed, with standard errors in parentheses. ENTUSE = ICT use at home for entertainment activities; HOMESCH = ICT use at home for schoolwork; USESCH = ICT use at school in general; USESCH2 = intermediated USESCH (the second classification of USESCH intensity, USESCH1=low USESCH as the reference group); USESCH3 = intensive USESCH; USESCH4 = excessive USESCH; READ=reading literacy; UR = Understanding and Remembering (one aspect of metacognition); SM = Summarizing (one aspect of metacognition); AC = Assessing credibility of information (one aspect of metacognition).

* $p < .05$. ** $p < .01$. *** $p < .001$.
